# A modified multiple cross displacement amplification linked with a gold nanoparticle biosensor for the detection of Epstein-Barr virus in clinical applications

**DOI:** 10.3389/fmicb.2023.1268572

**Published:** 2023-10-11

**Authors:** Xiaoyan Zeng, Xinggui Yang, Ludi Yang, Xu Yi, Xu Chen, Junfei Huang, Yu Wang, Shijun Li

**Affiliations:** ^1^The Second Affiliated Hospital, Guizhou University of Traditional Chinese Medicine, Guiyang, Guizhou, China; ^2^Guizhou Provincial Center for Disease Control and Prevention, Guiyang, Guizhou, China; ^3^Tongren People's Hospital, Tongren, Guizhou, China; ^4^Department of Clinical Laboratory, The First People's Hospital of Guiyang, Guiyang, Guizhou, China

**Keywords:** Epstein-Barr virus, multiple cross displacement amplification, gold nanoparticles-based lateral flow biosensor, EBV-MCDA-LFB, detection

## Abstract

Epstein-Barr virus (EBV), a double-stranded DNA virus belonging to the family *Herpesviridae*, infects more than 95% of healthy adults by attacking the host immune system. Here, a novel detection protocol, utilizing the modified multiple cross displacement amplification (MCDA) technique combined with a gold nanoparticles-based lateral flow biosensors (AuNPs-LFB), was devised and developed to detect EBV infection (termed EBV-MCDA-LFB assay). Ten MCDA primers targeting the *EBNA-LP* gene were designed, including CP1* primers modified with 6-carboxyfluorescein (FAM) and D1* primers modified with biotin. Then, nucleic acid templates extracted from various pathogens and whole blood samples were used to optimize and evaluate the EBV-MCDA-LFB assay. As a result, the lowest concentration of EBNA-plasmids, which can be detected by MCDA-LFB assay with an optimal reaction condition of 67°C for 30 min, was 10 copies/reaction. Here, the MCDA-LFB assay can detect all EBV pathogens used in the study, and no cross-reactions with non-EBV organisms were observed. Meanwhile, the entire detection workflow of the EBV-MCDA-LFB assay for whole blood samples, including DNA template preparation (25 min), EBV-MCDA amplification (30 min), and AuNPs-LFB-mediated validation (2–5 min), can be completed within 1 h. Taken together, the EBV-MCDA-LFB assay established in the current study is a rapid, simplified, sensitive, specific, and easy-to-obtain technique that can be used as a screening or diagnostic tool for EBV infection in clinical applications, especially in resource-poor regions.

## Introduction

Epstein-Barr virus (EBV), a double-stranded DNA virus belonging to the family *Herpesviridae*, was discovered in 1964 in suspension cultures of African Burkitt lymphoma cells (Odumade et al., 2011; Phaneuf et al., 2013). Globally, EBV infects more than 95% of healthy adults by attacking the host immune system, and infectious mononucleosis (IM) is most common in EBV-affected populations (Odumade et al., 2011; Liu et al., 2013). Although EBV-associated IM is a self-limiting disease, it occasionally causes serious complications including pneumonitis, airway obstruction, hemophagocytic syndrome, etc. (Iwata et al., 2006; Liu et al., 2013). Moreover, EBV is also a tumor-inducing causative agent, usually associated with the pathogenesis of many malignancies, such as Hodgkin’s lymphoma (HL), Burkitt’s lymphoma (BL), EBV-associated post-transplantation lymphoproliferative disorders (PTLD), and nasopharyngeal carcinoma (NPC; Middeldorp et al., 2003; Li et al., 2009; Saha and Robertson, 2011; Liu et al., 2013). Thus, early identification of EBV pathogens is an ideal strategy for the prevention and control of EBV-associated diseases.

Although diagnostic methods based on clinical presentation and serology can achieve the purpose of detecting EBV infection in clinical application, these techniques have self-drawbacks, including low sensitivity, poor specificity, and expensive testing costs (Iwata et al., 2006; Liu et al., 2013). In particular, serological test is difficult to fulfill the strategy for early detection because it requires a convalescent-phase serum collected from individual patients (Liu et al., 2013). Therefore, an ideal detection method for detecting EBV infections should have rapid, sensitive, specific, and low-cost characteristics, especially in resource-poor areas. Currently, PCR-based (polymerase chain reaction) tests like real-time fluorescence PCR are still used as conventional detection methods for detecting nucleic acid fragments of EBVs in clinical applications (Phaneuf et al., 2013; Tonoyan et al., 2020). The real-time PCR assay relies on real-time detection systems, making it challenging to perform in resource-limited laboratories; moreover, the detection time (~2 h) and workflow (multiple heating cycle) also need to be further improved to meet the requirements of rapid detection. As a result, the development of a timely, simplified, sensitive, reliable, and easily available detection technique is extremely important for the early diagnosis of EBV infection.

Recently, isothermal amplification techniques such as multiple cross displacement amplification (MCDA; Wang et al., 2015), loop-mediated isothermal amplification (LAMP; Notomi et al., 2000; Yang et al., 2022), recombinase polymerase amplification (RPA; Yang et al., 2023c), and cross-priming amplification (CPA; Wang et al., 2021) were devised and applied successfully in many pathogens detection. These isothermal techniques overcome the disadvantages of PCR-based assays that require a thermal cycler because they only require a single temperature (Yang et al., 2023a). The MCDA technique is a novel nucleic acid isothermal amplification technique first proposed and developed by Wang et al. in 2015 (Wang et al., 2015). Compared to the RPA and RPA-based assays, the amplification mechanism of the MCDA technique driven by a single polymerase is more straightforward, and its cost is lower (Lobato and O’Sullivan, 2018; Yang et al., 2022). In the MCDA reaction system, a set of MCDA primers, including two displacement primers (F1 and F2), six amplification primers (C1, C2, R1, R2, D1, and D2), and two cross primers (CP1 and CP2), can identify 10 regions of the objective sequence, thus showing high specificity for distinguishing target pathogens from non-target pathogens (Yang et al., 2022). However, conventional MCDA tests rely heavily on visualization reagents (i.e., hydroxy naphthol blue and SYBR Green) or agarose gel electrophoresis for product validation (Wang et al., 2015). Due to the approaches mentioned above being universal validation methods, it is not easy to accurately distinguish between specific and non-specific MCDA amplification. Hence, it is urgent to design a novel validation method to accurately verify MCDA amplicons.

With the application of nanomaterials in clinical diagnosis, gold nanoparticles-based lateral flow biosensors (AuNPs-LFB) utilizing the specific modification protocol with 6-carboxyfluorescein (FAM) and biotin can resolve the limitations (Yang et al., 2021a,b). That is, 10 MCDA primers, including the CP1* primer modified with FAM and D1* primer modified with biotin, produce FAM/target/Biotin amplicons exponentially in the presence of the *Bst* enzyme and target templates (Yang et al., 2022, 2023b). The AuNPs-LFB biosensor, pre-immobilized with fluorescein isothiocyanate (FITC) antibody as a test line, specifically captured FAM/target/Biotin-Streptavidin-AuNPs (Streptavidin-coated AuNP nanoparticles were captured by biotin as the FAM/target/Biotin amplicons flow the conjugate pad; Yang et al., 2022). In the AuNPs-LFB biosensor, the reaction mechanism similar to the antibody-binding antigen ensures the verification results’ reliability and specificity. Thus, the MCDA technique modified with FAM and biotin, combined with an AuNPs-LFB biosensor, is a proper and promising protocol for diagnosing EBV infection.

In this work, the modified MCDA technique based on AuNPs-LFB biosensor characterized by visualization, rapidness, sensitivity, and high specificity was devised and developed to detect the EBV virus (EBV-MCDA-LFB). In the EBV-MCDA-LFB reaction system, ten specific MCDA primers targeting the Epstein-Barr virus nuclear antigen leader protein (*EBNA-LP*) gene were designed according to the MCDA reaction principle. Here, various nucleic acid templates extracted from pathogens and whole blood samples were used to evaluate the diagnosis capability of the EBV-MCDA-LFB assay for EBV infection.

## Materials and methods

### Reagents and apparatus

Universal deoxyribonucleic acid isothermal amplification kits and visual MG reagents (malachite green) were purchased from Tian-Jin Huidexin Technology Development Co., Ltd. (Tianjin, China). Viral qEx-DNA/RNA extraction kits and universal bacterial genomic DNA extraction kits were obtained from Xi′an Tianlong Science &Technology Co., Ltd. (Xi′an, China). These biomaterials, including backing card, sample pad, conjugate pad, nitrocellulose membrane (namely, NC membrane), and absorbent pad were purchased from Jie-Yi Biotechnology. Co., Ltd. (Shanghai, China). Biotinylated bovine serum albumin (biotin-BSA) and rabbit anti-FITC antibody (anti-FITC) were obtained from Abcam. Co., Ltd. (Shanghai, China). Dye (crimson red) streptavidin-coated gold nanoparticles (SA-AuNPs) were provided by Bangs Laboratories, INC. (Indiana, USA). Real-time turbidimeter (*LA*-500) was provided by Eiken chemical Co., Ltd. (Japan). The ChemiDoc MP imaging system obtained from Bio-Rad (USA).

### Design and preparation of AuNPs-LFB biosensor

**Figure 1:**
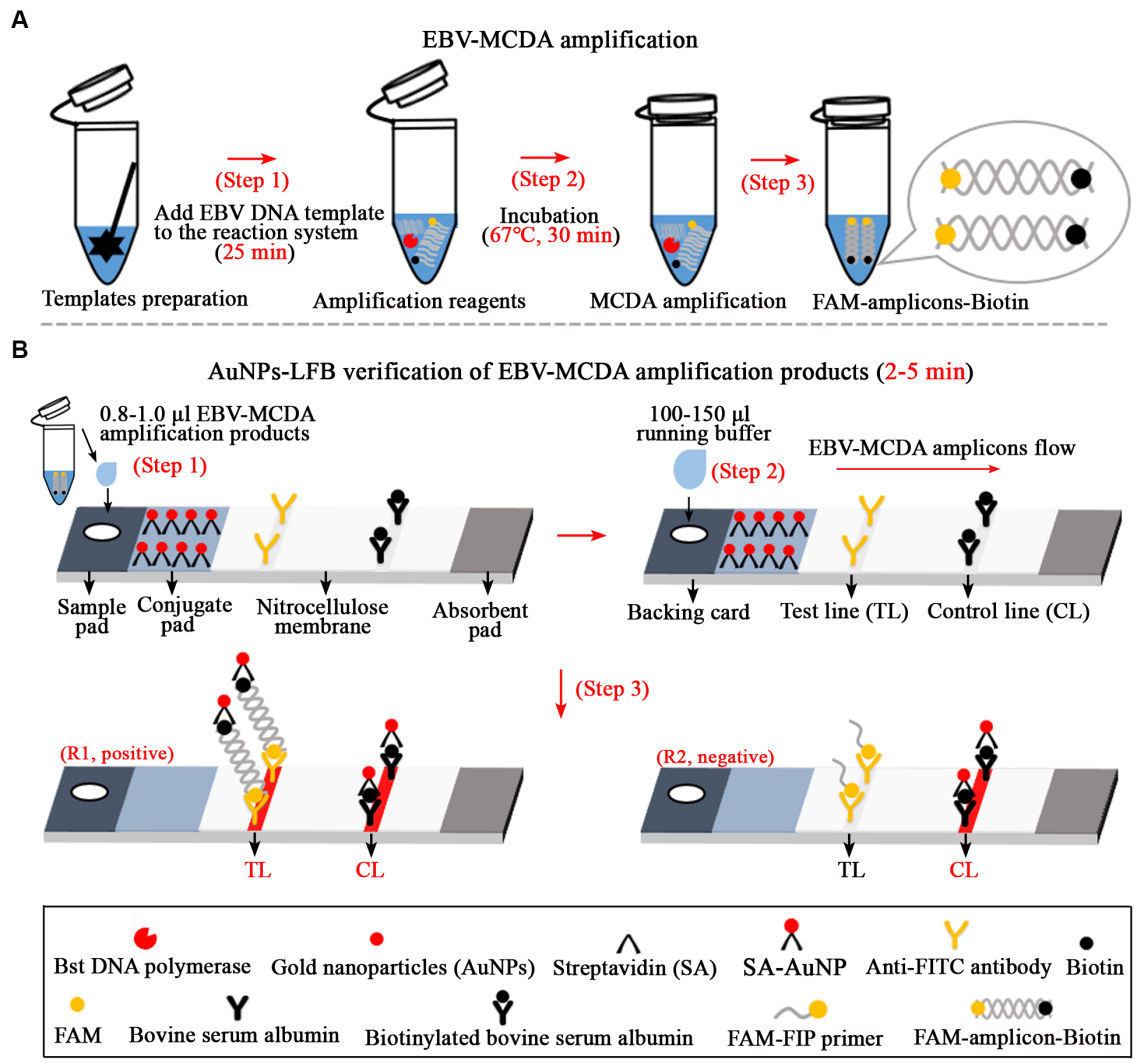
Overview of the detection workflow of the EBV-MCDA-LFB assay. The EBV-MCDA-LFB assay mainly includes rapid preparation of EBV DNA template (**A**, step 1), MCDA amplification in a 0.2-ml reaction tube premixed with reaction reagents and DNA templates (**A**, steps 2 and 3), and AuNPs-LFB-mediated validation for MCDA amplification (**B**, steps 1, 2, and 3). Here, the FAM-amplicons-biotin complex combined with SA-AuNPs (streptavidin-coated gold nanoparticles) in the conjugated pad can be captured by anti-FITC on the NC membrane to form a TL line. Meanwhile, the superfluous SA-AuNPs, driven by the running buffer, can be captured by biotin-BSA on the NC membrane to form CL lines. Finally, there are two test results on the AuNPs-LFB biosensor: EBV-positive (R1, both TL and CL lines are red) and EBV-negative (only CL line is red). MCDA, multiple cross displacement amplification; AuNPs-LFB, gold nanoparticles-based lateral flow biosensor; TL, test line; CL, control line.

The AuNPs-LFB biosensor was designed according to the principle of FAM’s specific binding to the anti-FITC antibodies (Li S. et al., 2020; Yang et al., 2022). The AuNPs-LFB biosensor is mainly composed of a sampling region, conjugate region, binding region (NC membrane) and absorbent region. A total of two lines separated by 5 mm, including the test line (TL) and the control line (CL), were constructed on the NC membrane of the AuNPs-LFB biosensor. Specifically, the rabbit anti-FITC antibody with a concentration of 0.15 mg/ml in 0.01 M PBS (pH 7.4) was deposited on an NC membrane close to the sample pad to form the TL line; other side, the biotinylated bovine serum albumin (biotin-BSA) with a concentration of 2.5 mg/ml in 0.01 M PBS (pH 7.4) was deposited to form the CL line. The SA-AuNP nanoparticles (129 nm, 10 mg ml^−1^, 100 mM borate, pH 8.5 with 0.1% BSA, 0.05% Tween 20, and 10 mM EDTA) were collected in a conjugate pad located in the middle of the sample pad and the NC membrane. Subsequently, these components, including the sample pad, conjugate pad, NC membrane, and adsorbent pad, were fixed on the backing card using plastic adhesive. According to our design, AuNPs-LFB biosensors are assembled by TianJin HuiDeXin Biotech. Co., Ltd. (Tianjin, China) and stored at room temperature (dry environment) away from light. The detection workflow (including the design principle) of the AuNPs-LFB biosensor for validating MCDA amplicons was shown in Figure 1.

### Design of specific primers and standard plasmids for EBV-MCDA-LFB assay

In this EBV-MCDA-LFB reaction system, five sets of specific MCDA primers targeted repetitive regions of the *EBNA-LP* gene (GenBank accession number, MK973061.1), including F1, F2, C1, C2, R1, R2, D1*, and D2, CP1*, and CP2 primers, were designed using the Primer Premier software (5.0). The sequence alignment of designed EBV-MCDA primers was performed using the BLAST software (basic local alignment search tool software). Subsequently, five sets of MCDA primers were synthesized to screen the optimal set of specific primers used in this study. According to the reaction principle of AuNPs-LFB biosensor, the FAM and biotin groups were engineered at the 5 ‘end of CP1* primer and D1* primer, respectively. Details of EBV-MCDA primers were shown in Table 1. The EBV-MCDA primers (HPLC purification grade) were synthesized by Tianyi-Huiyuan Biotech Co., Ltd. (Beijing, China). Moreover, the EBNA-plasmids used in this study were prepared and quantified by synthesizing *EBNA-LP* gene (GenBank accession number, MK973061.1), and subsequently cloning them into a pUC57 vector. The entire preparation process (i.e., cloning and extraction) was carried out by Tianyi-Huiyuan Biotech Co., Ltd. (Beijing, China). After quantification, the EBNA-plasmids with initial concentrations of 3 × 10^10^ copies were diluted to prepare a series of diluents (10^6^, 10^5^, 10^4^, 10^3^, 10^2^, 10^1^, 10^0^, and 10^−1^ copies).

**Table 1:** Primers used in this study.

Primers^a^	^b^Sequences and modifications (5′-3′)	Length^c^
EBNA-F1	AATAAGCCCCCAGACAGG	18 nt
EBNA-F2	CCCCTCTTACATTTGTGTGG	20 nt
EBNA-CP1*	FAM-CTAGCAACGCGAACCCCCTT-GGAGTGGGCTTGTTTGTGA	39 mer
EBNA-CP2	TTGTCAGTTCTAGGGAGGGGGA-TGATGCGACCAGAAATAGCT	42 mer
EBNA-C1	CTAGCAACGCGAACCCCCTT	20 nt
EBNA-C2	TTGTCAGTTCTAGGGAGGGGGA	22 nt
EBNA-D1*	Biotin-GCCCTGACCTTTGGTGAAG	19 nt
EBNA-D2	CACTGCCCCTGGTATAAAGTG	21 nt
EBNA-R1	AACGCGCTGGACTGAGAAGG	20 nt
EBNA-R2	TACGTAAGCCAGACAGCAGC	20 nt

a FAM, 6-Carboxyfluorescein.

b In our study, the conserved region targeting the EBNA-LP gene is homologous only to herpesvirus 4; meanwhile, homology with other Herpesviruses (e.g., 1, 2, 3, 5, 6, 7, and 8) does not exist in the NCBI nucleic acid database.

c nt, nucleotide; mer, monomeric unit.

### Preparation of nucleic acid templates

A total of 22 pathogens, including 11 viruses and 11 bacteria, were used to evaluate the specificity of the EBV-MCDA-LFB assay. These nucleic acid templates extracted from various organisms were prepared using the viral qEx-DNA/RNA extraction kits and universal bacterial genomic DNA extraction kits, respectively. All extraction steps are performed according to the manufacturer’s instructions. The prepared nucleic acid template is stored at −20°C and avoids repeated freeze–thaw. Details of the organisms, including names, sources, numbers, etc. were presented in Table 2. In addition, DNA templates were prepared from 107 whole blood samples to evaluate the EBV-MCDA-LFB assay. Briefly, after 1 ml of the whole blood sample is diluted with 0.9% NaCl solution, slowly add it to a glass test tube containing 500 μl of lymphocyte separation medium (Tianjin Haoyang Biological Manufacture Co., Ltd., Tianjin, China); and the tubes were centrifuged at 2,000 rpm for 10 min. Then, the leukocyte-rich solution was pipetted into a 1.5 ml centrifuge tube and centrifuged at 12,000 rpm for 5 min; then the supernatant was discarded, 50 μl of DNA extraction solution (Daan Gene Co., Ltd., Guangzhou, China) was added and mixed, and heated at 100°C for 8 min. Finally, the tubes were centrifuged at 12,000 rpm for 2 min, and the supernatant was stored at −20°C.

**Table 2:** Target or non-target organisms used in this study.

Organisms^a^	Source of strains^b^	No. of strains
Target organisms		
Inactivated EBV standard culture	BioBDS	1
Epstein-Barr virus	CHCIP	1
Non-target organisms		1
Human cytomegalovirus	BNCC	1
Dengue virus	CHCIP	1
Influenza B virus	CHCIP	1
Parainfluenza virus 1	CHCIP	1
Parainfluenza virus 3	CHCIP	1
Sendai virus	CHCIP	1
Vesicular stomatitis virus	CHCIP	1
Rubella virus	CHCIP	1
Coxsackievirus A16	CHCIP	1
*Brucella melitensis*	GZCDC	1
*Bacillus anthracis*	GZCDC	1
*Klebsiella Pneumoniae*	GZCDC	1
*Staphylococcus aureus*	GZCDC	1
*Streptococcus pneumoniae*	GZCDC	1
*Mycobacterium tuberculosis*	GZCDC	1
*Mycobacterium leprae*	GZCDC	1
*Haemophilus influenzae*	GZCDC	1
*Salmonellae* spp.	GZCDC	1
*Pseudomonas aeruginosa*	GZCDC	1
*Shigella* spp.	GZCDC	1
Total		22

a EBV, Epstein-Barr virus.

^b^CHCIP, Children’s Hospital Capital Institute of Pediatrics; BNCC, BeNa Culture Collection; GZCDC, Guizhou Provincial Center for Disease Control and Prevention.

### The EBV-MCDA-LFB reaction

The 25-μl amplification mixture of the EBV-MCDA assay contain the following: 12.5 μl of 2 × BF (reaction buffer), 1 μl of *Bst* thermostatic enzyme (2.0), 0.4 μM each of displacement primer (EBNA-F1 and EBNA-F2), 0.8 μM each of amplification primer (EBNA-C1, EBNA-C2, EBNA-R1, EBNA-R2, EBNA-D1*, and EBNA-D2), 1.6 μM each of cross primer (EBNA-CP1* and EBNA-CP2), 1 μl of MG reagent, 1 μl of nucleic acid templates extracted from pure culture and 2 μl of nucleic acid templates extracted from whole blood samples, and double-distilled water were added to 25 μl. The mixed system of EBV-MCDA assay was continuously amplified at 63°C for 60 min. Then, the amplification products of the EBV-MCDA assay were verified using an AuNPs-LFB biosensor, MG visualization indicator, 1.5% agarose gel electrophoresis, and real-time turbidimeter (*LA*-500). In addition, a one-microliter DNA template extracted from human cytomegalovirus was used as a negative control. One microliter of environment sample was used as a laboratory internal control. One microliter of double-distilled water was used as a blank control.

### Optimization of amplification conditions for EBV-MCDA-LFB assay

In the current study, the amplification conditions of the EBV-MCDA-LFB assay mainly included amplification temperature and time. Generally, amplification temperature, as a critical factor that can affect amplification efficiency, needs to be optimized in EBV-MCDA-LFB tests. Optimization tests of the EBV-MCDA assay were performed to explore the optimal incubation temperature. One microliter of EBNA-plasmid was used as the amplification template. The mixed tubes of the EBV-MCDA assay were incubated using different reaction temperatures (62–70°C with 1°C intervals) according to the EBV-MCDA-LFB reaction system. Then, the amplicons of the EBV-MCDA assay were validated using a real-time turbidimeter. In addition, an optimization tests of reaction time for the EBV-MCDA assay were implemented using 1 μl of each diluent of EBNA-plasmids as a template (10^6^, 10^5^, 10^4^, 10^3^, 10^2^, 10^1^, and 10^0^ copies). According to the EBV-MCDA reaction system, this time-optimized test was performed by setting different reaction times (10–60 min, 10 min intervals). Finally, the amplicons of the EBV-MCDA assay were verified using an AuNP-LFB biosensor.

### Evaluation test of sensitivity for EBV-MCDA-LFB assay

To evaluate the performance of the MCDA-LFB assay for the detection of low-concentration EBV-DNA templates, the sensitivity test was performed according to the optimal reaction system. One microliter of series diluents of EBNA-plasmids (10^6^, 10^5^, 10^4^, 10^3^, 10^2^, 10^1^, 10^0^, and 10^−1^ copies) was used as MCDA amplification templates. Next, the MCDA amplification products were validated using AuNPs-LFB biosensors, MG visualization reagents, 1.5% agar-gel electrophoresis, and a real-time turbidimeter. Finally, the lowest concentration that can be detected by the MCDA-LFB test after more than three repetitions are defined as its limit of detection (LoD).

### Evaluation test of specificity for EBV-MCDA-LFB assay

Here, 25 nucleic acid templates extracted from 22 pathogens and three EBV-positive whole blood samples were examined to evaluate the detection specificity of the MCDA-LFB assay (Table 2). According to the optimal MCDA reaction condition, 1 μl nucleic acid template was used as the detection object for MCDA-LFB test. Then, the MCDA amplicons of specificity tests were detected using AuNPs-LFB biosensors.

### Evaluation test of clinical applicability for EBV-MCDA-LFB assay

In the current study, 107 whole blood samples collected from patients with suspected EBV infection were used to evaluate the detection applicability of MCDA-LFB assay. The whole blood samples were prepared for DNA template using a commercial extraction kit, and all extraction procedures were followed by the kit’s instructions (as mentioned earlier). In order to better evaluate the detection performance of the MCDA-LFB assay, a conventional real-time PCR assay was used as a reference method. The MCDA-LFB assay was implemented based on the MCDA reaction system and optimal reaction conditions, and then the amplicons were validated using the AuNPs-LFB biosensors. Meanwhile, the real-time PCR assay was performed according to the commercial kits. Five microliters of DNA prepared from whole blood samples were used as amplification templates for MCDA-LFB and real-time PCR assays. Finally, the results of the real-time PCR assay were used as criteria to evaluate the applicability of the MCDA-LFB assay to detect whole blood samples with EBV infection.

## Results

### The working principle of the EBV-MCDA-LFB assay

**Figure 2:**
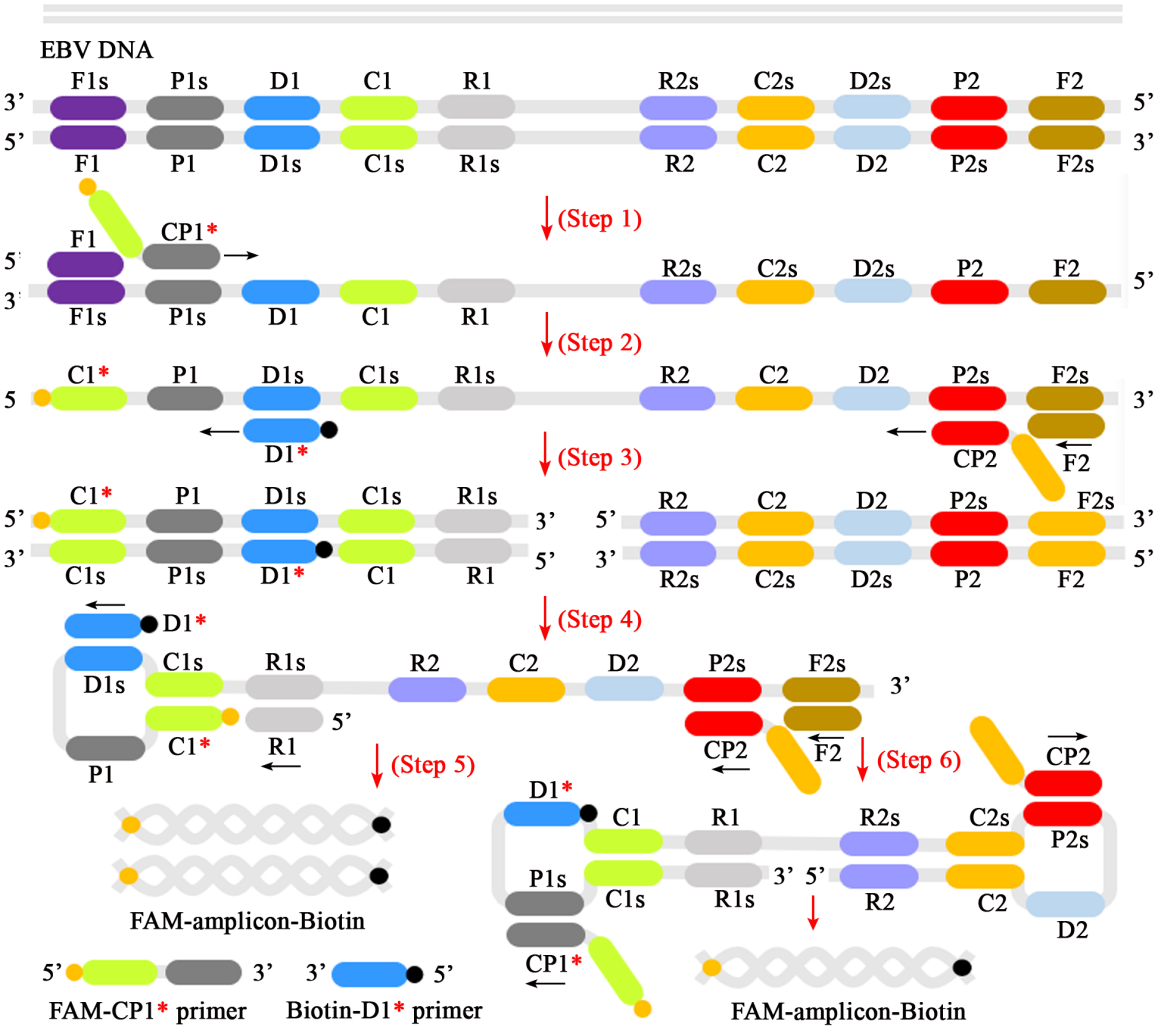
Schematic diagram of reaction mechanism of EBV-MCDA amplification. One set of MCDA primers (including CP1*, CP2, C1, C2, P1, P2, D1*, D2, R1, R2) can recognize 10 different regions on the target sequence (Step 1). The prepared double-stranded DNA of EBV was amplified specifically in the presence of *Bst* polymerase and MCDA primers. Then, the CP1* primer labeled with FAM can combine with complementary P1s site on the target and extends to synthesize new FAM-labeled double-stranded amplicons (Step 2). Subsequently, the D1* primer labeled with biotin binds to the complementary D1s site on the newly synthesized MCDA amplicons contained FAM group and extends further (Step 3). As a result, the exponentially amplified FAM-amplicons-biotin products were produced in the reaction mixture (Steps 4, 5, and 6). MCDA, multiple cross displacement amplification; AuNPs-LFB, gold nanoparticles-based lateral flow biosensor; FAM, 6-carboxyfluorescein.

In the MCDA-AuNPs-LFB reaction system, one set of MCDA primers can recognize 10 different complementary regions on the target (P1s, P2s, C1s, C2s, P1s, P2s, D1s, D2s, R1s, R2s; Figure 2, step 1). The prepared double-stranded DNAs of EBV were amplified specifically in the presence of *Bst* DNA polymerase and MCDA primers. That is, the CP1* primer labeled with FAM can combine with complementary P1s site on the target and extends to synthesize new FAM-labeled double-stranded amplicons (Figure 2, step 2). Subsequently, the D1* primer labeled with biotin binds to the complementary D1s site on the newly synthesized MCDA amplicons contained FAM group and extends further (Figure 2, step 3). As a result, the exponentially amplified FAM-amplicons-biotin products were produced in the MCDA reaction mixture (Figure 2, steps 4, 5, and 6). In other words, the whole detection process, including preparation of the EBV template (Figure 1A, step 1), reaction reagents premixing and MCDA amplification (Figure 1A, steps 2 and 3), and AuNPs-LFB-mediated validation of EBV-MCDA amplicons (Figure 1B, steps 1, 2, and 3), can be completed in a short time (within 60 min). In the AuNPs-LFB detection system, the FAM-amplicons-biotin complex combined with SA-AuNPs (streptavidin-coated gold nanoparticles) in the conjugated pad can be captured by anti-FITC on the NC membrane to form a TL line (Figure 1B, R1). Meanwhile, the superfluous SA-AuNPs, driven by the running buffer, can be captured by biotin-BSA on the NC membrane to form CL lines to verify the effectiveness of AuNPs-LFB biosensors (Figure 1B, R1/R2).

### Assessment test of the EBV-MCDA-LFB assay

**Figure 3:**
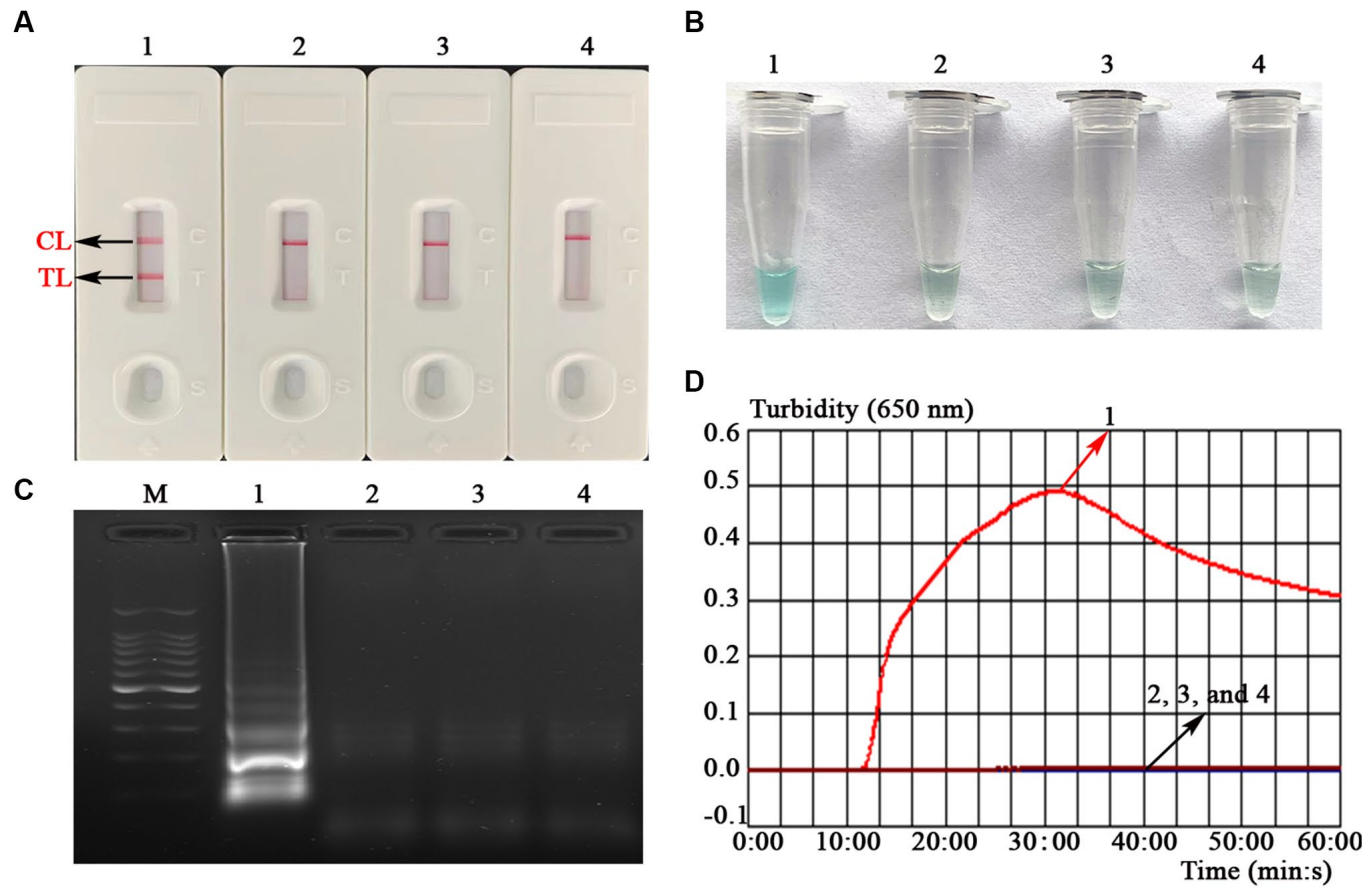
Assessment assay of the EBV-MCDA-LFB assay. Amplicons of EBV-MCDA were validated using AuNPs-LFB biosensor **(A)**, MG visualization reagent **(B)**, 1.5% agarose gel electrophoresis **(C)**, and real-time turbidimeter **(D)**. Biosensor **(A1)**/tube **(B1)**/lane **(C1)**/line **(D1)**, positive amplification of the EBV-MCDA assay using the EBNA-plasmids; Biosensor **(A2)**/tube **(B2)**/lane **(C2)**/line **(D2)**, negative control of the EBV-MCDA assay using the nucleic acid template extracted from human cytomegalovirus; Biosensor **(A3)**/tube **(B3)**/lane **(C3)**/line **(D3)**, negative control of the EBV-MCDA assay using the environmental sample in the experiment; Biosensor **(A4)**/tube **(B4)**/lane **(C4)**/line **(D4)**, negative control of the EBV-MCDA assay using double-distilled water. Lane M, DL100 DNA Marker; MCDA, multiple cross displacement amplification; AuNPs-LFB, gold nanoparticles-based lateral flow biosensor; TL, test line; CL, control line.

To verify the feasibility of the MCDA-LFB assay designed in the current study, a confirmation test was performed based on the EBV-MCDA reaction system. One microliter EBNA-plasmids with a concentration of 10^6^ copies was used as an amplification template. Next, the amplification results were validated using the four validation methods (i.e., AuNPs-LFB biosensor, MG amplification indicator, 1.5% agarose gel electrophoresis, and real-time turbidimeter). As expected, when AuNPs-LFB biosensor was used to verify the EBV-MCDA results, both TL and CL regions were red in the positive results (Figure 3A); In contrast, only the CL region was red, and the TL region was colorless for negative reactions (Figure 3A). After adding the MG amplification reagent to the reaction system, it can be observed by the naked eye that the positive reaction mixture is blue, and the negative reaction mixture is light blue or colorless (Figure 3B). The MCDA amplification products were visualized by staining with ethidium bromide in a 1.5% agarose gel under ultraviolet light, and the positive results showed bright bands with characteristic gradients (Figure 3C). In addition, MCDA amplification products can also be monitored by real-time turbidimeters; usually, the turbidity threshold is set to 0.1; greater than 0.1 is considered positive amplification (Figure 3D).

### Optimal performance conditions of the EBV-MCDA-LFB assay

**Figure 4:**
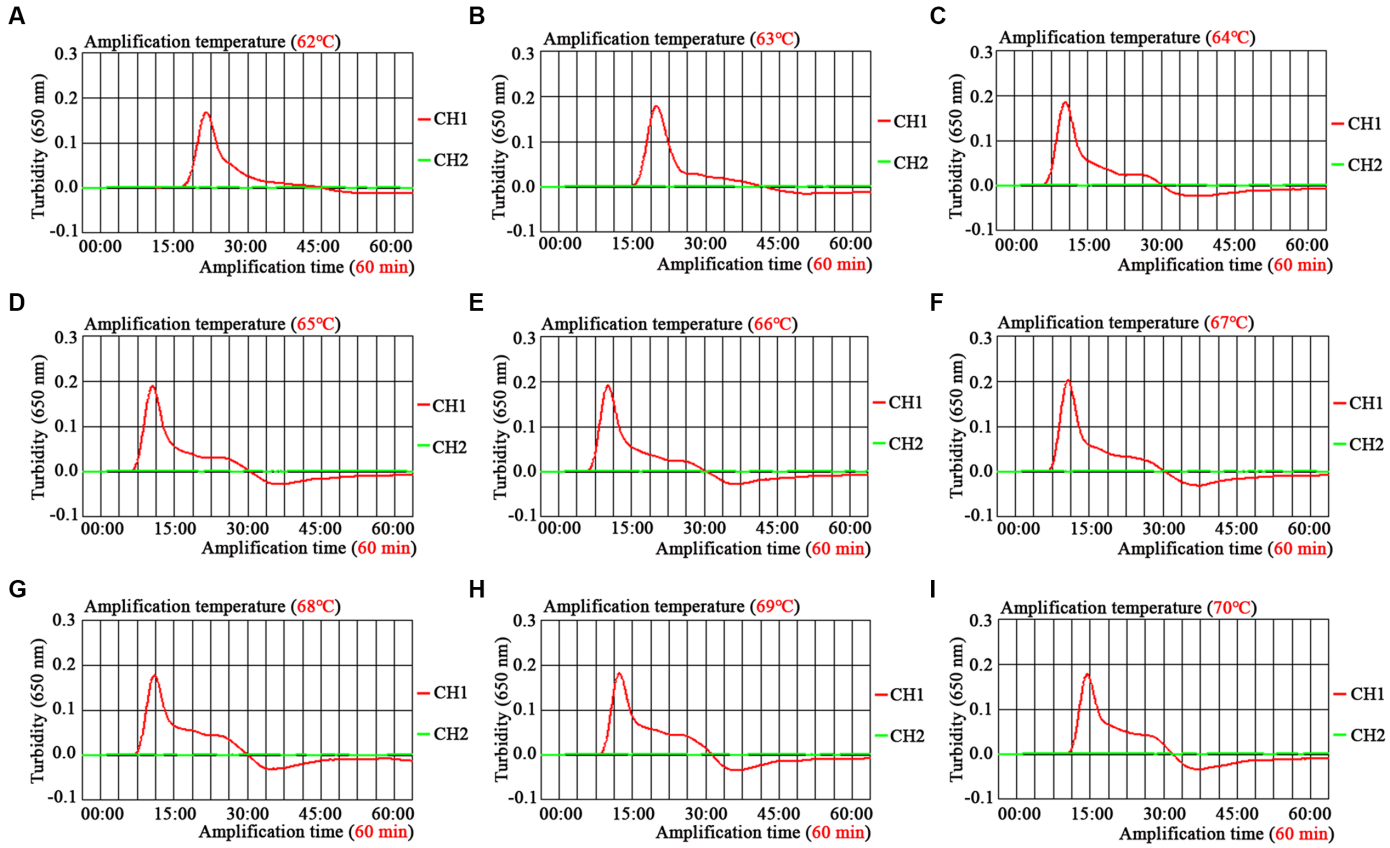
Temperature optimization of optimal primers for EBV-MCDA amplification. A total of nine dynamic curves were obtained using different amplification temperatures ranging from 62 to 70°C with 1°C intervals **(A–I)**. According to the default setting of the real-time turbidimeter (*LA*-500), the threshold was 0.1; the turbidity value greater than 0.1 was judged as positive amplification; on the contrary, the turbidity value less than 0.1 is negative. The amplification efficiency of EBV-MCDA is better than other temperatures when the amplification temperature is 67°C **(F)**. MCDA, multiple cross displacement amplification.

**Figure 5:**
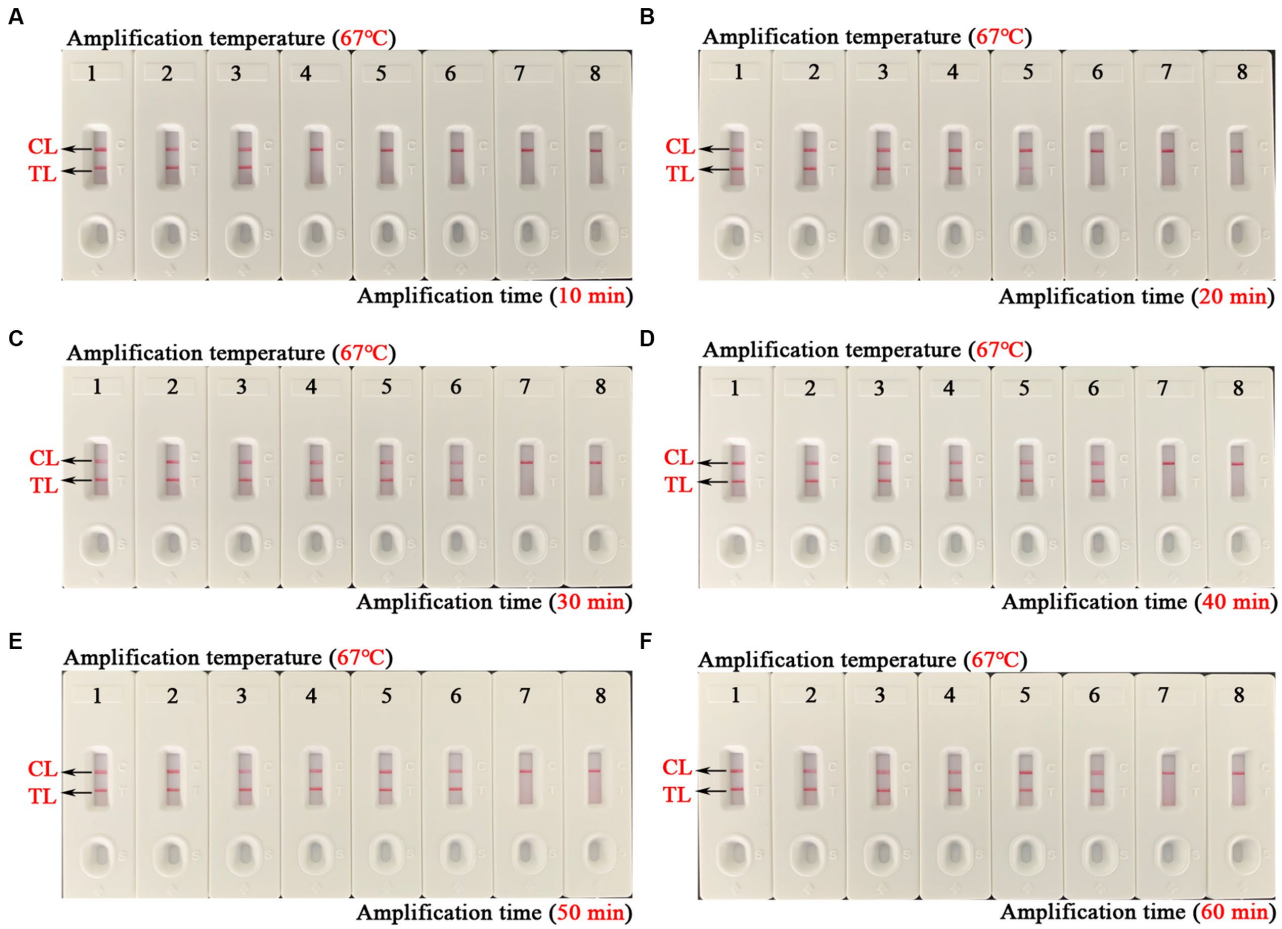
Time optimization of optimal primers for EBV-MCDA-LFB assay. The optimal reaction time of EBV-MCDA-LFB was optimized by using a series of diluents of EBNA-plasmids as an amplification template (i.e., 10^6^, 10^5^, 10^4^, 10^3^, 10^2^, 10^1^, and 10^0^ copies). The combined drawings **(A–F)** correspond to time gradients ranging from 10 to 60 min with intervals of 10 min. Strips 1–7 **(A–F)** correspond to 10^6^, 10^5^, 10^4^, 10^3^, 10^2^, 10^1^, and 10^0^ copies. Biosensors 8 **(A–F)** correspond to blank control (double-distilled water). When the reaction time ranged from 30 to 60 min, the detection limit of the EBV-MCDA-LFB assay was 10 copies/reaction. Therefore, the optimal reaction time of the EBV-MCDA-LFB was 30 min in the study **(C)**. MCDA, multiple cross displacement amplification; AuNPs-LFB, gold nanoparticles-based lateral flow biosensor; TL, test line; CL, control line.

The optimal amplification temperature was explored by setting a series of temperature gradients (62–70°C, 1°C intervals) to improve the detection efficiency (Figure 4). In the current study, when the reaction temperature was 67°C, the amplification efficiency of the EBV-MCDA assay was the highest by observing the real-time dynamic drawings of real-time turbidity (Figure 4F). In addition, amplification time is an essential factor that needs to be optimized to achieve rapid detection of EBV. The lowest concentration of a series of diluted EBNA-plasmids (10^6^, 10^5^, 10^4^, 10^3^, 10^2^, 10^1^, and 10^0^ copies) that the EBV-MCDA-LFB assay can detect was 10 copies when tested against the reaction time of 30, 40, 50, and 60 min (Figure 5). Thereby, the optimal amplification time of the EBV-MCDA-LFB assay was found as 30 min (Figure 5C). According to the above optimization tests, the optimal amplification condition of EBV-MCDA-LFB was 67°C for 30 min in the current study.

### Detection sensitivity of the EBV-MCDA-LFB assay

**Figure 6:**
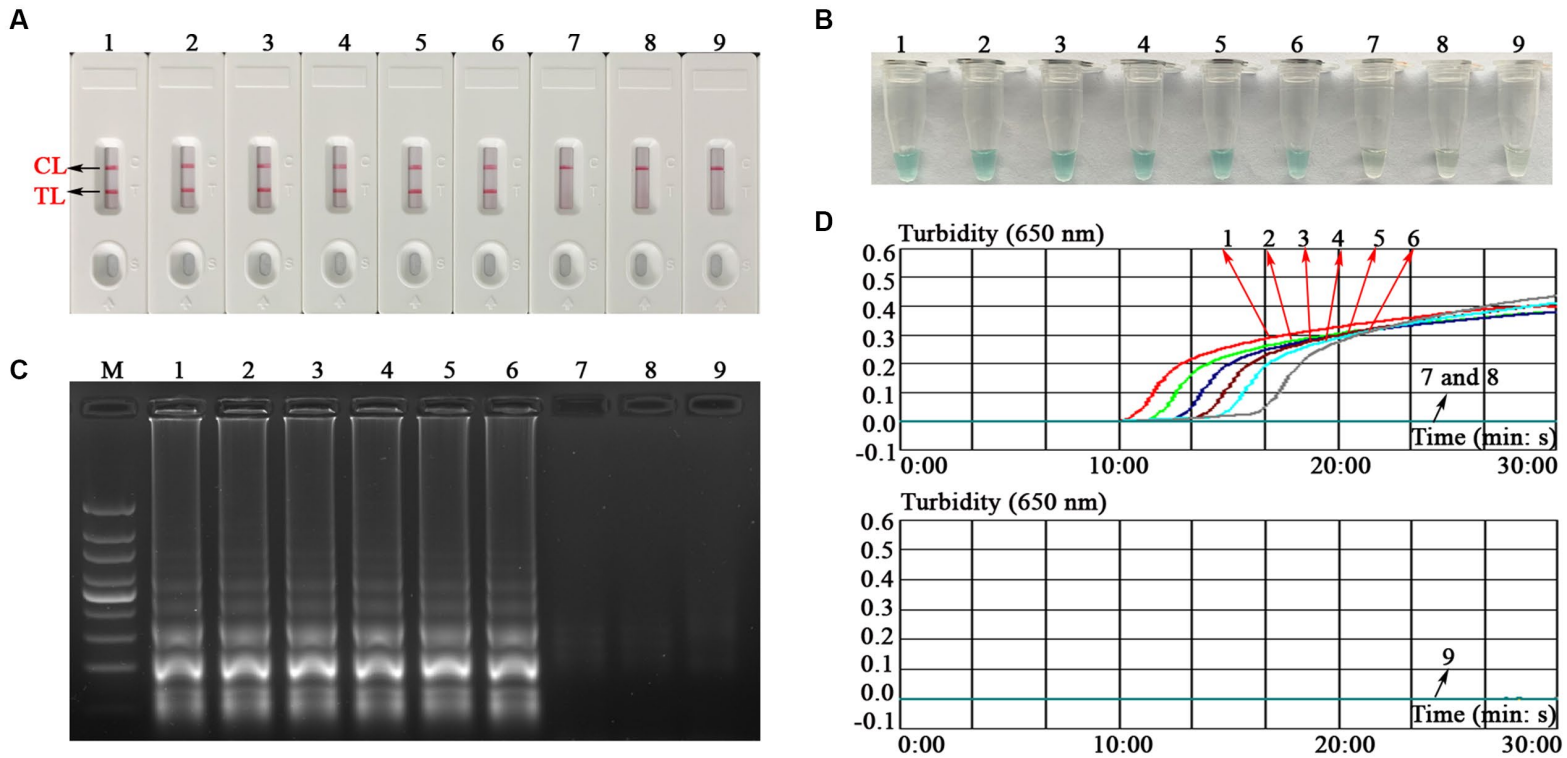
Sensitivity test of the EBV-MCDA-LFB assay. The sensitivity of EBV-MCDA-LFB was tested using different concentrations of EBNA-plasmids, and these amplification products were validated using AuNPs-LFB biosensor **(A)**, MG chromogenic reagents **(B)**, 1.5% agarose gel electrophoresis **(C)**, and real-time turbidimeter **(D)**. Biosensor **(A1–A8)**/tube **(B1–B8)**/lane **(C1–C8)**/line **(D1–D8)** correspond to 10^6^, 10^5^, 10^4^, 10^3^, 10^2^, 10^1^, 10^0^, and 10^−1^ copies. Biosensor **(A9)**/tube **(B9)**/lane **(C9)**/curve **(D9)** correspond to blank control (nuclease-free water). Lane M, 100 bp DNA ladder; MCDA, multiple cross displacement amplification; AuNPs-LFB, gold nanoparticles-based lateral flow biosensor; TL, test line; CL, control line.

To explore the ability of the EBV-MCDA-LFB assay to detect EBV templates with low concentrations, this sensitivity test was performed using a series of diluents of 1 microliter EBNA-plasmids as templates. The LoD of the EBV-MCDA-LFB assay was 10 copies/reaction after repeated trials (Figure 6).

### Detection specificity of the EBV-MCDA-LFB assay

**Figure 7:**
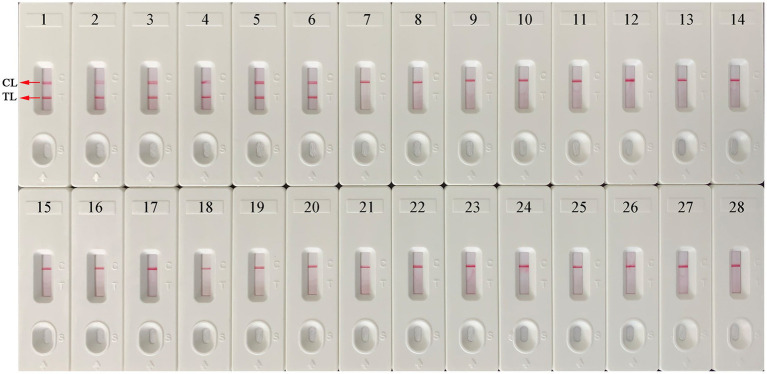
Specificity test of the EBV-MCDA-LFB assay. The specificity of EBV-MCDA-LFB was tested using various pathogens, and these amplicons were validated using AuNPs-LFB biosensors. Biosensor 1, EBV (inactivated standard EBV culture); Biosensor 2, EBV-template; Biosensor 3, EBNA-plasmids, Biosensors 4–6, EBV-positive clinical samples; Biosensors 7–15, Human cytomegalovirus, Dengue virus, Influenza B virus, Parainfluenza virus 1, Parainfluenza virus 3, Sendai virus, Vesicular stomatitis virus, Rubella virus, Coxsackievirus A16; Biosensors 16–26, *Brucella melitensis*, *Bacillus anthracis*, *Klebsiella Pneumoniae*, *Staphylococcus aureus*, *Streptococcus pneumoniae*, *Mycobacterium tuberculosis*, *Mycobacterium leprae*, *Haemophilus influenzae*, *Salmonella* spp., *Pseudomonas aeruginosa*, and *Shigella* spp.; Biosensors 27–28, negative control (environmental sample in the test) and blank control (double-distilled water). MCDA, multiple cross displacement amplification; AuNPs-LFB, gold nanoparticles-based lateral flow biosensor; TL, test line; CL, control line.

A total of 25 nucleic acids extracted from different pathogens and EBV-positive samples were used as amplification templates to evaluate the specificity of the EBV-MCDA-LFB assay (Table 2). As shown in Figure 7, the EBV-MCDA-LFB assay can detect all representative EBV organisms, including inactivated EBV standard culture (BioBDS), EBV (CHCIP), EBNA-plasmids, EBV-positive clinical samples, and can exclude other non-EBV-pathogens. That is, the detection specificity of the EBV-MCDA-LFB assay is 100% in the current study.

### Clinical applicability of the EBV-MCDA-LFB assay

Here, a total of 107 whole blood samples were used to evaluate the clinical applicability of the EBV-MCDA-LFB assay by comparing it with the results of the real-time PCR assay. Thirty whole blood samples (30/107) were tested as EBV-positive, and 77 were EBV-negative utilizing the real-time PCR assay (Table 3). Similarly, 30 clinical specimens tested as EBV-positive by real-time PCR assay were also detected as EBV-positive by the EBV-MCDA-LFB assay, and 77 were EBV-negative (Table 3). The positive rate of EBV-MCDA-LFB and real-time PCR was consistent in the current study. Of note, the MCDA-LFB has excellent detection capability for whole blood samples with different viral loads, including samples with lower viral loads quantified by real-time PCR assay (e.g., 532 copies, 589 copies, 778 copies, 967 copies, etc.). In general, these data confirm that the MCDA-LFB can be used as a competitive potential detection technique for EBV infection in clinical settings.

**Table 3:** Analysis of the results of using EBV-MCDA-LFB and real-time PCR assays to detect 107 whole blood samples.

Methods^a^	Real-time PCR		Total
	Positive	Negative	
EBV-MCDA-LFB			
Positive	30	0	30
Negative	0	77	77
Total	30	77	107

a PCR, polymerase chain reaction; MCDA, multiple cross displacement amplification; LFB, gold nanoparticles-based lateral flow biosensor.

## Discussion

The EBV, as a pathogen with a high infection rate and possibly a variety of diseases (e.g., IM, HL, BL, PTLD, and NPC), needs to be diagnosed early in EBV-affected populations (Iwata et al., 2006; Liu et al., 2013; Tonoyan et al., 2020). At present, real-time PCR technology, as a commonly used method to detect EBV, plays a crucial role in clinical detection. Undeniably, the real-time PCR assay has high sensitivity and specificity in the detection field of infectious diseases like SARS-Cov-2, *Mycobacterium tuberculosis*, etc. (Pitetti et al., 2003; Sales et al., 2014; Li D. et al., 2020). However, it is challenging to meet the demand for fluorescent thermal cycles required by real-time PCR technique in basic laboratories in resource-poor areas. Therefore, an efficient detection technique should also have easy availability (i.e., simple instrumentation requirements) in addition to its high sensitivity and specificity, especially in resource-limited regions.

Isothermal amplification-based assays seem to be able to meet the requirement above-mentioned, like MCDA, which is a more specific and sensitive assay than LAMP assays due to its ability to recognize 10 specific regions of the target sequence (Yang et al., 2021b, 2022). Currently, MCDA and MCDA-based techniques are widely used to detect various pathogens (Li S. et al., 2020; Yang et al., 2022, 2023b). However, the validation of amplification products has been the focus of the MCDA technique. A suitable identification method should hold these specific characteristics, such as convenience, speed, sensitivity, specificity, and accessibility in general laboratories. Hence, the AuNPs-LFB biosensor was designed according to the amplification principle of the MCDA in the current study (Yang et al., 2022). AuNP-LFB biosensor, as an efficient validation method, can effectively reduce the incidence of false positives in test results resulting from non-specific amplification recognized by conventional methods (such as visualization reagents, real-time turbidity, etc.). Importantly, the validation process takes less time (2–5 min) than conventional agar-gel electrophoresis (~1 h), resulting in the entire MCDA-LFB detection process being completed in a very short time (Figure 1). In addition, we designed a control line at the distal end of the AuNPs-LFB biosensor using the biotin-BSA complex to ensure the reliability of test results, especially negative results (Figure 1B).

As demonstrated in the confirmation test, the validation results of the AuNPs-LFB biosensor were consistent with the other three methods (e.g., MG visualization reagent, agarose gel electrophoresis, and real-time turbidity; Figure 3). This phenomenon proved the stability and reliability of the AuNPs-LFB biosensors for the verification of MCDA amplicons. In general, the selection of target genes is the key to MCDA detection. Thus, the *EBNA-LP* gene, one of the first viral genes expressed upon B-cell infection consisting of W1W2 repeats and a unique C-terminal Y1Y2 domain, is used to design MCDA primers (Kawaguchi et al., 2000; Szymula et al., 2018). However, the carryover contamination caused by the open-tube detection using AuNPs-LFB biosensors cannot be ignored due to the high amplification efficiency of the MCDA assay (Yang et al., 2022). Thus, it is necessary to strictly divide the experimental zones, including the sample preparation zone, the premixing zone of amplification mixtures, the MCDA amplification zone, and the amplicon validation zone. Overall, the strategy implemented above significantly reduce the possibility of carryover contamination in the EBV-MCDA-LFB assay.

In the sensitivity test, the lowest concentration of series diluents of EBNA-plasmids detected by MCDA-LFB assay was 10 copies/reaction, which is more sensitive than conventional real-time PCR assay (~500 copies; Figure 6). Meanwhile, the validation results of the AuNPs-LFB for MCDA amplicons were in agreement with MG visual reagents, 1.5% agar-gel electrophoresis, and real-time turbidimeter, confirming its reliability in practicality tests. The high sensitivity of the MCDA-LFB assay is necessary to improve the detectable rate, especially for EBV-infected whole blood specimens with low viral load in clinical applications. Moreover, the MCDA-LFB assay demonstrated excellent specificity, which can recognize all representative EBV pathogens [including, inactivated EBV standard culture (BioBDS), EBV (CHCIP), EBV-positive clinical samples] and EBNA-plasmids used in the study (Figure 7; Table 2). Importantly, no cross-reactions were observed here with all of the non-EBV pathogens used in the current study (including related pathogens like human cytomegalovirus). However, although no homology was found between the conserved regions of MCDA primers targeting the *EBNA-LP* gene and other herpesviruses, it must be acknowledged that due to limitations in the source of other herpesviruses, this study used only partial representative herpesvirus organisms (e.g., Epstein-Barr virus and Human cytomegalovirus) for specificity validation. Thus, more herpesvirus pathogens may need to be used for further validation in the future if necessary. In summary, these data confirm that the EBV-MCDA-LFB established is a highly sensitive and specific competitive detection protocol for identifying organisms used in this study.

In this study, the EBV-MCDA-LFB assay had reliable detection capability for EBV infection in clinical detection. Interestingly, the detectable rate of EBV-MCDA-LFB was consistent with that of the real-time PCR assay, confirming its high reliability in detecting EBV infection (Table 3). Undeniably, the inability to achieve quantitative detection is also a limitation of the EBV-MCDA-LFB assay; still, due to the different purposes of tests in clinical applications, quantitative detection is not necessary. Of note, the EBV-MCDA-LFB is more practical than a real-time PCR assay because the amplification process requires only a simple heating device, even a thermostatic water bath. As a result, these data demonstrate that the EBV-MCDA-LFB assay is a competitive potential detection technique for EBV infection in basic laboratories in clinical applications, especially in resource-poor areas. Meanwhile, the entire detection process of the EBV-MCDA-LFB assay for whole blood samples, including DNA template preparation (25 min), EBV-MCDA amplification (30 min), and AuNPs-LFB-mediated validation (2–5 min), can be completed within 1 h. Compared with real-time PCR assay (the detection workflow is about 2 h in this study), the EBV-MCDA-LFB assay can significantly shorten the detection time (~50%), which is of great practical significance for achieving rapid detection. In addition, the cost for detecting a single whole blood sample using EBV-MCDA-LFB assay can be controlled within 6.0 USD, which is lower than the real-time PCR assay (~25 USD). Based on the data mentioned above, the MCDA-LFB assay established in the current study is a rapid, simple, low-cost, highly sensitive, and specific assay that can be used as a screening or diagnostic assay for EBV infection in clinical settings.

## Conclusion

In the current study, a novel detection protocol utilizing the designed AuNPs-LFB biosensor combined with MCDA amplification (termed EBV-MCDA-LFB assay) was developed and implemented to detect EBV infection in clinical settings. The EBV-MCDA-LFB assay demonstrated outstanding detection efficiency for EBNA-plasmids, EBV-standard culture, EBV-positive clinical samples, and clinical whole blood samples. Thus, the MCDA-LFB assay established in the current study is a fast, simple, low-cost, highly sensitive, and specific assay that can be used as a screening or diagnostic assay for EBV infection in clinical settings. Taken together, the EBV-MCDA-LFB assay established in the current study is a fast, simplified, sensitive, specific, and easy-to-obtain technique that can be used as a screening or diagnostic tool for EBV infection in populations, especially in resource-limited regions.

## Data availability statement

The original contributions presented in the study are included in the article/supplementary material, further inquiries can be directed to the corresponding author.

## Ethics statement

This study was approved by the Human Ethics Committee of the Second Affiliated Hospital of the Guizhou University of Traditional Chinese Medicine (Ethical Approval No. KYW20230827) and complied with the Declaration of Helsinki.

## Author contributions

XZ: Conceptualization, Data curation, Methodology, Software, Validation, Writing – original draft, Writing – review & editing. XYa: Conceptualization, Data curation, Methodology, Software, Validation, Writing – original draft, Writing – review & editing. LY: Writing – original draft. XYi: Methodology, Supervision, Writing – original draft. XC: Methodology, Writing – original draft. JH: Methodology, Writing – original draft. YW: Methodology, Writing – original draft. SL: Supervision, Conceptualization, Formal analysis, Funding acquisition, Investigation, Project administration, Resources, Visualization, Writing – review & editing, Writing – original draft.
